# Technical guidance on biodegradation testing of difficult substances and mixtures in surface water

**DOI:** 10.1016/j.mex.2023.102138

**Published:** 2023-03-20

**Authors:** Heidi Birch, Rikke Hammershøj, Mette Torsbjerg Møller, Philipp Mayer

**Affiliations:** a Technical University of Denmark, Department of Environmental and Resource Engineering, Bygningstorvet, Building 115, 2800 Kgs, Lyngby, Denmark; b Technical University of Denmark, Department of Environmental Engineering, Bygningstorvet, Building 115, 2800 Kgs, Lyngby, Denmark

**Keywords:** Difficult-to-test substances, Closed test design, Solvent free testing, Whole substance testing, Aerobic Simulation Biodegradation Test of Difficult Substances in Closed Vials

## Abstract

The aim of this article is to address critical challenges in the OECD 309 “Aerobic mineralization in surface water – simulation biodegradation test” for volatile chemicals, highly hydrophobic chemicals, mixtures or UVCBs (unknown or variable composition, complex reaction products or biological materials). Several modifications are presented to address technical challenges (minimize and account for losses), make testing more environmentally relevant (lower concentrations) and generate data for multiple substances (more and better aligned data):

• **Minimizing and accounting for test substance losses:** Aqueous solutions are handled using gas tight syringes, tests are conducted in gas tight vials, and automated analysis is performed directly on unopened test vials. Abiotic losses are accounted for via concentration ratios between test systems and abiotic controls that are incubated and measured in parallel.
• **Testing at low environmentally relevant concentrations:** Substances are tested at low concentrations to avoid toxicity and solubility artefacts and analyzed using a sensitive analytical method. Substances are added without co-solvent (using passive dosing) or with a minimum of co-solvent (using microvolume spiking).
• **Testing of multiple chemicals in mixtures combined with constituent specific analysis:** Primary biodegradation kinetics of chemicals are determined in tests of multi-constituent mixtures or UVCBs using constituent specific analysis.

## Graphical abstract

**Figure:**
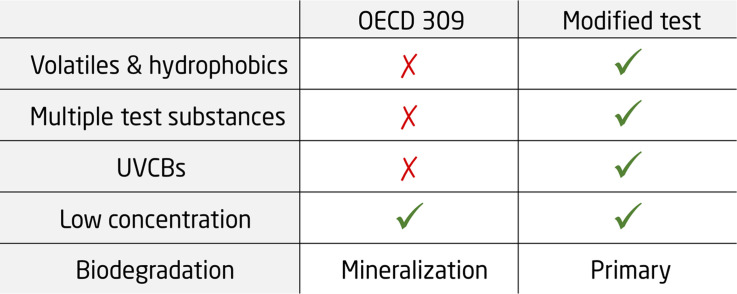

Specifications table

**Table:** 

Subject area:	Environmental Science
More specific subject area:	Biodegradation testing
Name of your method:	Aerobic Simulation Biodegradation Test of Difficult Substances in Closed Vials
Name and reference of original method:	Birch, H., Andersen, H., Comber, M., Mayer, P. Biodegradation testing of chemicals with high Henry's constants – separating mass and effective concentration reveals higher rate constants. Chemosphere, 2017, Vol. 174, p. 716-721. Birch, H., Hammershøj, R., Mayer, P. Determining Biodegradation Kinetics of Hydrocarbons at Low Concentrations: Covering 5 and 9 Orders of Magnitude of Kow and Kaw. Environ. Sci. Technol, 2018, Vol. 52, p. 2143-2151 Hammershøj, R., Sjøholm, K.K., Birch, H., Brandt, K.K., Mayer, P. Biodegradation kinetics testing of two hydrophobic UVCBs – potential for substrate toxicity supports testing at low concentrations. Environmental Science Processes & Impacts, 2020, Vol. 22, p. 2172-2180. Møller, MT., Birch, H., Sjøholm, K.K., Hammershøj, R., Jenner, K., Mayer, P. Biodegradation of an essential oil UVCB – Whole substance testing and constituent specific analytics yield biodegradation kinetics of mixture constituents. Chemosphere, 2021, Vol. 287, p. 130409. OECD 309, OECD Guideline for the testing of Chemicals. Aerobic Mineralization in Surface Water – Simulation Biodegradation Test, 2004.
Resource availability:	N.A.

## Method overview

Biodegradation testing is important for assessing the persistence of organic chemicals in the environment. The OECD guidelines describe a tiered approach for this, going from simple screening tests to simulation biodegradation tests [1,2]. The standard simulation biodegradation tests are however not applicable for difficult to test chemicals such as volatile chemicals, highly hydrophobic chemicals, mixtures or UVCBs (unknown or variable composition, complex reaction products or biological materials) [3]. The aim of this paper is to present test modifications that are necessary to make the OECD 309 “Aerobic mineralization in surface water – simulation biodegradation test” better suited for volatile chemicals, hydrophobic chemicals, mixtures and UVCBs. Our recommendations are based on test method improvements that have been made in a number of recent research studies [4], [5], [6], [7], [8], [9]. We hope that this tutorial method paper can help to (1) apply our methods at other laboratories, (2) facilitate technical improvements of other methods and possibly even (3) serve as input for an OECD guidance document for the aquatic biodegradation testing of difficult test chemicals. Such an OECD guidance document has been published and recently updated for the aquatic toxicity testing of difficult test chemicals [10] and a similar guidance document is urgently needed in the field of biodegradation testing [11].

The primary challenge when testing volatile chemicals is evaporative losses from test systems during set-up, incubation, and sampling. This applies not only to substances with high vapor pressure and low boiling point, but also to higher boiling substances with high air-water partition coefficients. For non-labeled test chemicals, such losses can be mistaken for primary biodegradation (removal of parent chemical), and the extent of biodegradation overestimated. For ^14^C-labeled chemicals, losses can be tracked through measurements of residual radioactivity in the test. In actively aerated systems, liquid or solid-state traps can also be used to determine evaporative losses and improve mass balances. However, considerable losses of test substances can in any case prevent the determination of meaningful degradation kinetics, i.e., if evaporative loss rates are considerably faster than degradation rates. For volatile chemicals, it is therefore necessary to minimize evaporative losses, include appropriate controls and account for minor evaporative losses. It is crucial to use closed test systems without aeration during incubation, and these systems should ideally not be opened before extraction/analysis. These constraints point towards a biodegradation methodology where test systems are prepared in gas tight vials that are compatible with an autosampler, and where analysis is performed directly on the unopened test systems.

The primary challenges when testing hydrophobic chemicals are low water solubility and sorptive losses. A low water solubility requires a low test concentration to ensure that chemicals are fully dissolved. If a pure phase of the test chemical is introduced in the biodegradation test, the biodegradation rate may be limited by dissolution kinetics rather than by the ability of the inoculum to degrade the chemical. At concentrations close to solubility, hydrophobic chemicals may also inhibit the degrader organisms. It is therefore important to test the biodegradation of hydrophobic chemicals at concentrations well below their water solubility. The low test concentrations lead to challenges for the analytical determination of the chemicals. Solid Phase Microextraction (SPME) coupled to GC/MS has proven a sensitive analytical method for hydrophobic test chemicals because the SPME fiber enriches the hydrophobic chemicals according to their hydrophobicity. To minimize sorptive losses, it is necessary to avoid plastic materials in contact with aqueous solutions of the hydrophobic test chemicals during test set up and incubation. If chemicals are volatile and hydrophobic, plastic materials should not be in contact with the headspace either. This points to the use of glass and PTFE as materials in test systems. It also necessitates the use of abiotic control systems to account for any losses from test systems.

For mixtures and UVCBs, the challenge is to test the complete mixture and gain information on the degradation of individual components [3,12]. While the main focus of the OECD 309 is to determine the mineralization kinetics in surface water [2], other relevant endpoints are also targeted i.e. primary and ultimate degradation and transformation products. For single test chemicals ^14^C-labeling of the most recalcitrant part of the chemical structure is important to determine mineralization rather than primary degradation of the chemical. With mixtures this becomes much more complicated, and it is often not possible to ^14^C-label UVCBs. Further, the measurement of ^14^CO_2_ evolution does not reveal which constituents degrade and which do not. The focus on primary biodegradation kinetics is thus more relevant for mixtures. This can be obtained by chemical specific analysis of the constituents in mixtures and main constituents of UVCBs. In this way, it is possible to evaluate the biodegradation of UVCBs on a constituent basis and screen for possible persistent (major) constituents.

## General principles of the test

**Fig. 1:**
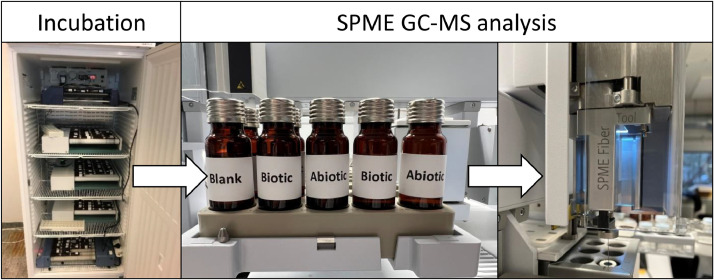
Method principle: A large number of biotic and abiotic test systems are prepared in headspace vials, incubated at constant temperature, and analyzed using automated sorptive enrichment by solid phase microextraction directly coupled to GC/MS. .

To address the numerous challenges outlined above for biodegradation testing of (semi)volatile and hydrophobic chemicals in mixtures or UVCBs, we propose a method that is based on a close alignment between the testing in gas tight headspace vials and the automated sorptive enrichment by Solid Phase Microextraction (SPME) directly coupled to GC/MS analysis (see Fig. 1). Hydrophobic and (semi)volatile chemicals are added to natural water containing native degrader microorganisms and incubated at a fixed temperature, in the dark, under aerobic conditions, and with continuous agitation. Special attention is given to minimize evaporative losses, sorptive losses, and the use of co-solvents during the test setup. Abiotic test systems without microorganisms are incubated in parallel to account for abiotic losses during the test. In line with the OECD 309 test, the test is conducted with low environmentally relevant chemical concentrations (<1 µg/L – 100 µg/L), which implies that the test chemicals can serve as secondary substrates and be degraded by co-metabolism [2]. At pre-determined time points, three biotic and three abiotic test systems are taken for analysis. To avoid opening the vials, each test system can be extracted with SPME through the septum. The SPME is coupled to e.g., GC–MS, for chemical specific analysis. Primary biodegradation is then determined for each mixture constituent from the ratios between the analytical signal in the biotic test system relative to the signal in the abiotic test system.

## Applicability of the test

The test method extends the applicability domain of the OECD 309 and is applicable to mixtures of hydrophobic and volatile chemicals. The method is developed as a pelagic test used to simulate biodegradation in surface water free of coarse particles.

The applicability of the method has been demonstrated for very volatile chemicals with high Henry´s constants [4], mixtures of hydrocarbons that covered a wide chemical space in volatility and hydrophobicity [5], and UVCBs [8]. It has also been used for determining the mixture effect, the temperature effect and the concentration effect on the biodegradation kinetics of a wide range of chemicals [9,^13^,14].

## Information on test substances

The required information on the test substance is not very different from the current OECD 309 recommendations, however, the importance and use of each property may vary. In short, a minimum amount of information on the test substances is required to choose the most appropriate test method setup. This includes solubility in water, volatility as expressed by Henry's laws constant, hydrolysis/stability in water, and n-octanol/water partition coefficient. If experimental values are missing, (Q)SAR based estimates may be used [15,16]. Other valuable information includes solubility in solvents, dissociation constant, and toxicity of test substances towards microorganisms [2].

## Reference substances

It is possible to add reference substances to the test mixture. The role of these reference substances is to confirm that the surface water contains an active microbial population and is not inhibited. These chemicals might also serve as benchmark chemicals when comparing data in modified test methods. The reference substances should be analyzable using the same analytical method as the test chemicals. In our latest project we have used naphthalene and phenanthrene as reference substances since they were included in many of our previous experiments and their degradation was seen to be sensitive towards the density of cultivable bacteria in the surface water used as inoculum [14]. Degradation half-times for naphthalene were in the previous experiments generally within 3–7 days for active surface waters, while degradation did not occur in water with low densities of cultivable bacteria [4,^5^,^14^,17].

## Quality criteria

Volatile and hydrophobic chemicals are, due to abiotic losses, at risk of not meeting the quality criteria on targeted recovery in the guideline OECD 309 (70–110% recovery at the end of the experiment based on mass balance). When working with non-labeled test chemicals a mass balance approach to evaluate the recovery is not possible. Instead, analysis of abiotic test systems at each measurement point can verify the extent of abiotic losses. Peak area ratios between biotic and abiotic test systems can then be used to evaluate biodegradation while correcting for these losses. It is not possible to determine biodegradation kinetics of chemicals if abiotic loss processes are faster than the biodegradation. In such cases the analytical response in abiotic controls may decrease below detection limits. The inclusion of standards during analysis can reveal the kinetics of abiotic losses but is not required for determination of biodegradation.

The test should be aerobic throughout the incubation. The validity of the test should therefore be demonstrated through the measurement of dissolved oxygen or through another justification for aerobic conditions (i.e. initial oxygen in test vs theoretical oxygen demand of all organic carbon in test).

Criteria for repeatability and sensitivity of the analytical method does not differ from those described in the OECD 309 [2].

## Description of test method

### Equipment

The main requirement on test system format for non-volatile hydrophobic chemicals is that glass test bottles should be used. If semi-volatile or volatile test chemicals are included, test systems should be closed and gas tight. Repeated opening of test systems for sub-sampling is not appropriate, as this may induce test substance losses. A practical solution to this is to use relatively small, closed test systems (20 mL) that are analyzed by destructive sampling instead of large test systems that are subsampled. The disadvantage of using smaller test systems is that there may be larger variation between biodegradation in test systems if rare microbes are involved. This is mainly a problem if inoculum with low bacterial densities are used [5,17]. One advantage is however, that each vial is a true replicate test system, and variability between test systems thus accounted for in the determination of biodegradation kinetics. Another advantage is that automated analysis on unopened test systems is possible.

Semi-volatile test chemicals (Henry's law constants, K_H_, between 10^−5^ and 10^−3^ atm m^3^ mol^−1^) can in most cases easily be handled in closed test systems without major losses [2]. For volatile test chemicals (K_H_ > 10^−3^ atm m^3^ mol^−1^), special attention should be given to the type of lid and septum used. The authors found that septa can vary significantly in how well they prevent losses – even for septa with the same specifications in terms of materials (e.g., PTFE / Silicone). The authors have found smaller losses when using white PTFE / transparent blue silicone septa compared with red PTFE / white silicone septa.

In addition to this, necessary equipment includes:

- An incubator or temperature-controlled room that can be operated at the chosen incubation temperature and is large enough to hold an appropriate number of roller mixers
- Roller mixers, enough to accommodate the anticipated number of test systems
- Gas tight glass syringes for transfer of solutions if working with volatile test chemicals and the passive dosing method
- Automated micro-syringes if microvolume spiking is chosen as dosing method
- Instrumentation for a suitable analytical method e.g., GC–MS, with automated solid phase microextraction

### Collection, transport, storage, and preparation of surface water

The type and origin of the surface water inoculum is very important for biodegradation tests. Inoculum from five locations varied more than one order of magnitude in heterotrophic plate count and in biodegradation half-time for each chemical (8 hydrocarbons) [17]. Lower degradation was seen in inocula with lower heterotrophic plate counts and less pre-exposure in terms of wastewater treatment plant effluent or separate sewer discharges. To create transparency regarding test inoculum, it is therefore important to report geographic origin, type of inoculum and further characterize the inoculum by measuring e.g., total suspended solids, dissolved organic carbon, nutrients (in case of seawater), and selected microbial parameters.

Surface water should be sampled and transported to the laboratory as fast as possible. The OECD 309 guideline recommends cooling to 4 °C if transport exceeds 2–3 h. However, storage and incubation temperature has a clear effect on the microbial composition of samples [18]. We therefore recommend that the water is kept at sampling or test temperature during transport and short storage (preferably < 1 day).

### Selection of test concentrations

Selection of test chemical concentrations is not trivial for hydrophobic chemicals, since conflicting considerations affect the optimal concentration level: 1) environmental relevance, 2) analytical detection level, 3) solubility/toxicity limitations and 4) oxygen demand. Analytical detection should be sufficient to quantify at least 10% of initial test substance concentration to reliably determine degradation rates. Solubility of test chemicals sets the upper limit for test chemical concentrations used, since dissolution of test chemicals may otherwise interfere with the measurements of degradation kinetics. At concentrations close to solubility, hydrophobic chemicals may inhibit degraders. Test concentrations should therefore not be too close to solubility. Further, it should be checked that the oxygen demand of the test chemicals does not exceed the oxygen present in the test system (See Eq. (1) or OECD 301 for an elaboration on the calculation of oxygen demand). This is especially relevant when testing mixtures with many constituents or UVCBs, where it is crucial to keep the oxygen demand of the entire mixture well below the oxygen present in the test system.

### Addition of test substances

Hydrophobic chemicals are challenging to add to test systems since their low water solubility prevents the preparation of spike solutions in water. Losses of test chemicals from spike solutions in water can also be high and unpredictable during storage and handling.

The most traditional way to handle hydrophobic chemicals is to use solvent spiking. This method is recommended in the OECD 309 guideline [2], where it is advised to use a volatile solvent that is stripped off before test start. This is however not possible for volatile and hydrophobic test chemicals as they would then also be lost. The OECD 305 [19] allows a ‘solid phase desorption dosing system’ to deliver chemicals to fish in bioaccumulation tests, but does not describe the method.

**Fig. 2:**
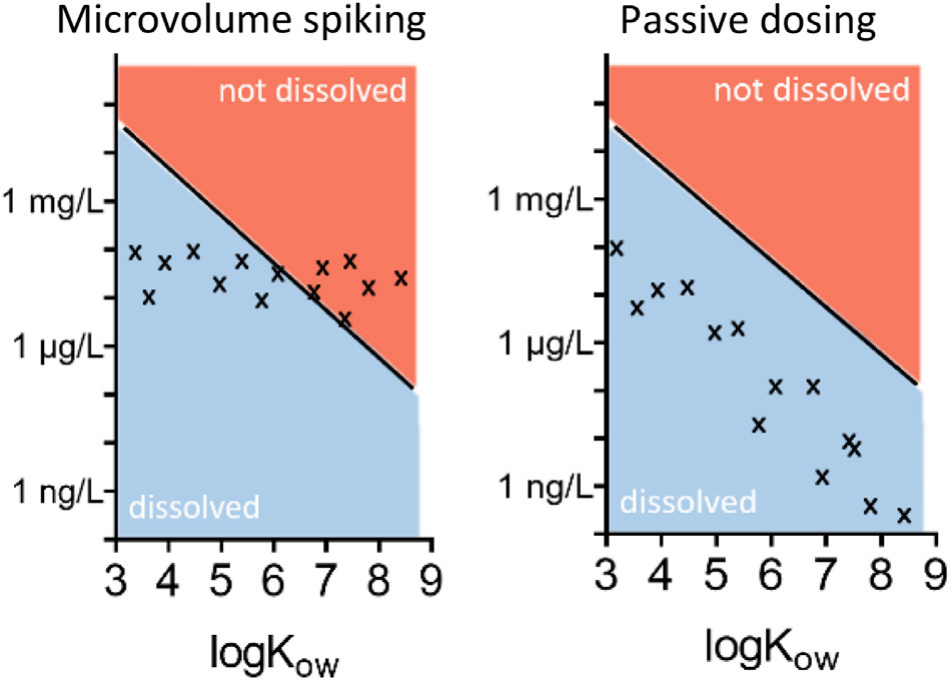
Illustration of the two dosing principles 1) quantitative transfer of mixtures via microvolume spiking (left) and 2) partitioning based transfer of chemicals via passive dosing from e.g., silicone rods (right). Concentration of mixture constituents and their octanol-water partition coefficient (LogK_ow_). Black line indicates water solubility. Figure modified from [20].

We here describe two different solutions for the addition of hydrophobic and volatile mixtures. The first is to use passive dosing for a completely solvent free addition of test chemicals to test systems. The second is to use microvolume spiking to minimize the effect of the solvent as much as possible. There are fundamental differences between the two methods as described in Hammershøj et al. 2020 [6]. Whereas microvolume spiking is based on the quantitative transfer of chemicals to test systems, passive dosing is based on partitioning of chemicals from a dominant donor into water. This means that microvolume spiking ensures that the composition of a mixture is transferred to test systems unchanged. Passive dosing, on the other hand, modifies the composition based on the hydrophobicity of the mixture constituents. While this may at first sound inappropriate for testing of chemical mixtures, it is highly environmentally relevant. When a mixture is released to the environment below the water solubility of its constituents, the composition will within a short time be modified due to partitioning to organic matter, sediments, and soil. This means that the more hydrophobic chemicals will be present at lower dissolved concentrations in the environment. A very convenient advantage of the passive dosing method is that chemicals are not inadvertently added at concentrations above their water solubility. Fig. 2 illustrates the differences between the two dosing methods.

#### Passive dosing

Passive dosing is applicable for chemicals with a log octanol-water partition ratio (logK_ow_) above 3. For these chemicals the passive dosing donor, e.g., PDMS (silicone), can be designed to act as a dominating partitioning phase with negligible depletion during use. This means that the donor will produce stable freely dissolved concentrations of test chemicals in water in equilibrium with the donor even at very low concentrations. Depending on the volumes of donor and water and on the hydrophobicity of the test chemicals, passive dosing systems can potentially be reused many times.

In our biodegradation test set-up, passive dosing systems are primarily used to prepare a spike solution in pure water, which is then transferred to test systems using gas tight syringes. It is also possible to add inoculum directly to the passive dosing systems in order to produce water with higher test chemical concentrations [14]. When doing so, it is important to minimize the equilibrium time for the passive dosing, since biodegradation will then start during dosing. Also, it is not advisable to reuse passive dosing systems if inoculum is added to the passive dosing systems because of possible bacterial contamination of the second batch of water.

For more information on the preparation and loading of passive dosing systems, see Hammershøj et al. 2020 [6].

#### Microvolume solvent spiking

Microvolume solvent spiking is feasible with handheld automated syringes that allow transferring 1–5 µL volumes much more precisely than manual syringes. The method has the practical advantage over passive dosing that the preparation before test setup is less time consuming. As illustrated in Fig. 2, it is important to be aware of the hydrophobicity of the mixture constituents to avoid precipitation of test chemicals. Further, it requires careful planning to avoid oxygen depletion in test systems.

The oxygen use for degradation of solvents is not a problem when tests are performed in open test systems. However, it is a major issue in closed test systems. This was for example clearly seen in an OECD 308 test (simulation biodegradation in water-sediment systems), in a study by Shrestha et al. [21], where biofilm formation was observed on top of the water phase and oxygen levels dropped fast when solvents were used for spiking. Simple estimates of the oxygen level in the test system and the oxygen demand of the added volume of solvent can help determine the appropriate test design. It is important to note that the concentration of oxygen in air is approximately 35 times higher than in water. The headspace in test systems is therefore very important to ensure sufficient oxygen levels throughout the test. Agitation of test systems, e.g., rolling or shaking, ensures that oxygen from the headspace can enter the water phase when the oxygen in the water is used for degradation.

To evaluate the appropriate water to headspace ratio, calculations of the amount of oxygen in the headspace can be compared to calculations of the theoretical oxygen demand of the solvent. The theoretical oxygen demand with nitrification for a chemical C_c_H_h_Cl_cl_N_n_Na_na_O_o_P_p_S_s_ with a molecular weight, MW, can be calculated according to Eq. (1) [2]. This is the maximum amount of oxygen needed for degradation.

(1) $$ThOD_{NO3}=\frac{32c+8h-8cl+40n+48s+40p+8na-16o}{MW}mg\;O_{2}/mg$$

The first time this method was used, 15 mL surface water was added to 20 mL headspace vials – leaving a 5 mL headspace [4]. The oxygen in a 5 mL headspace amounts to 1.4 mg O_2_. Since methanol has a theoretical oxygen demand of 1.5 mg O_2_ / mg methanol (Eq. (1)), the degradation of 1 µL methanol in this system would nearly deplete the oxygen in the headspace. In the work by Møller et al. [8], test system dimensions were therefore changed to a reduced water volume of 10 mL leaving 10 mL headspace, and a spike volume of 1 µL methanol was used. In cases where surface water or wastewater treatment plant effluent is used as inoculum in tests, the solvent will be the most dominant source of oxygen depletion. It should however be noted that some inocula may contain other degradable compounds that require oxygen for degradation, and care should be taken if e.g., activated sludge is used in tests.

### Test conditions

#### Test temperature

Incubation of test systems are done at a constant temperature. As recommended in the OECD 309, the temperature can be set at the relevant field temperature or standard temperatures such as 20 or 12 °C. In two studies, a clear effect was found on biodegradation kinetics of petroleum hydrocarbons at different incubation temperatures [9]. This effect was consistent with the Arrhenius equation when water sampled at low field temperatures (2.7 and 12 °C) were incubated at higher temperatures, and when water sampled at high field temperatures (17 °C ) were incubated at slightly lower temperatures (12 °C) [9]. However, when surface water sampled at 17 °C was incubated at 2.7 °C, nearly no degradation was seen [18]. The incubation temperature also impacts the physical-chemical properties of the test chemicals. This means that at lower concentrations the evaporative losses decrease due to lower henry's law constants, while the risk of precipitation of hydrophobic chemicals increases due to lower solubility. The most appropriate test temperature can be determined from the considerations: If the aim is to determine biodegradation kinetics at the specific site, then it is optimal to incubate at field temperature; If the aim is to compare degradation kinetics between chemicals and tests, then a standard temperature is optimal.

#### Agitation

Agitation is recommended during incubation to ensure that microbes are kept in suspension and biofilm formation, which can alter biodegradation kinetics during testing, is avoided. Since test systems in this method is closed during incubation, rolling or soft shaking are both good options. While magnetic stirring is allowed in the OECD 309, magnetic stirrers are not recommended here, since it is not possible to remove the magnets before analysis (performed on unopened test systems).

#### Test duration

The standard test duration for simulation biodegradation testing is up to 60 days. Test duration can be prolonged if extra test systems are prepared during test setup. It should however be noted that long test durations are less likely to reveal biodegradation kinetics that are relevant for the surface water of interest, since long lag phases may be caused by extensive adaptation of the microbial population [22,23].

Extra test systems can be prepared for analysis of oxygen at certain time points if this is deemed necessary based on the choice of test chemical concentrations or the content of organic matter in the inoculum.

## Procedure

### Preparation of test systems

Surface water inoculum is collected as specified above, and 15 mL is transferred to all biotic test systems prepared in 20 mL headspace vials, leaving 5 mL headspace. If necessary, the headspace can be adjusted to increase the initial amount of oxygen in the test systems (may be relevant for microvolume spiking). If passive dosing is used, up to 10% spike solution can be used, and the water volume is adjusted accordingly.

Abiotic controls are prepared in the same way as biotic test systems, except that pure water is used instead of surface water. Using pure water is appropriate if the surface water has a relatively low amount of suspended solids and dissolved organic matter [9]. Filter sterilized abiotic controls are needed if the inoculum contains considerable amounts of dissolved organic matter, since the solubility and volatility of the chemicals could then be affected. If the inoculum contains considerable amounts of particles, and sorption to these is expected to be high, then autoclaved or poisoned abiotic controls can be prepared as described in the OECD 309.

Spiking is done using gas tight syringes (passive dosing) or automated syringes (microvolume spiking), that are emptied below the water surface in the test system. Each vial should be closed immediately after addition of test chemicals to avoid losses of volatile test chemicals.

Test systems are then incubated (see section on incubation), and at each measurement time the pre-selected number of biotic and abiotic test systems are transferred to the autosampler for analysis. Appropriate measurement times cannot be stated universally as biodegradation kinetics is highly variable from chemical to chemical and depends on the type of surface water used. Also, mixtures may include chemicals that degrade at very different rates.

If no sample conservation is used, biodegradation in the test systems will continue up until the analysis. If incubation is done at low temperatures and the autosampler is not temperature controlled, test vials should not be left at the autosampler for extended time periods, since temperature highly affects the degradation kinetics of test chemicals [9]. This is more important for the short incubation times, where the time on the autosampler constitutes a higher fraction of the total incubation time compared to later measurements.

### Number of test systems

The number of test systems should be determined based on the desired number of measurement times and replicates. Duplicate or triplicate test systems can be prepared for each measurement time to evaluate the uncertainty between test systems. It is also possible to measure one set of biotic and abiotic test systems at each measurement time, but then increase the number of measurement times. An equal number of biotic and abiotic test systems should be prepared as well as some blank test systems and test systems with non-spiked surface water for quality control. Reference substances can be included in the biotic and abiotic test systems along with the test chemicals, and do not need separate test systems.

### Analysis

Solid Phase Microextraction (SPME) coupled to GC–MS is applicable for many hydrophobic and volatile chemicals. The SPME fiber and GC–MS column materials can be optimized based on the specific test chemicals. The SPME extraction can be done in the headspace or as immersion SPME depending on the volatility of the test chemicals. When test systems are analyzed alternating biotic and abiotic test systems (see Fig. 1), a potential drift in the analytical signal is accounted for. It should be noted that degradation below the analytical carryover limit cannot be detected. Careful consideration of the cleaning steps of the SPME fiber between samples may be required to lower the analytical carryover for very hydrophobic chemicals.

## Data and reporting

### Data treatment

#### Biotic/abiotic ratios

The use of abiotic test systems for reference, allows the biodegradation to be determined without analyzing standards at each measurement point. Instead, the following procedure can be used: 1) The linearity of the analytical signal is verified by standards before the initiation of the test, 2) external calibration of the initial test concentration is performed at test start, 3) at each timepoint 1–3 sets consisting of an abiotic test system and a biotic test system is analyzed, 4) the decrease in concentration stemming from biodegradation is determined from the peak area ratio between the biotic test system and abiotic test system. These peak area ratios correct for drift in analytical signal from time point to time point as well as possible abiotic losses. Thus, this is a simple and robust way to directly determine the amount of test chemical that has been biodegraded at a certain point in time.

Quality assurance includes a careful integration of the analytical peaks and a determination of the analytical detection limit. The analytical carryover (e.g., from the SPME fiber) can be measured from a number of standards and subsequent blank samples, and the cutoff can be determined as mean plus 3 times standard deviation on these ratios [14]. When full degradation occurs, some of the ratios will be below either the detection limit or the carryover limit (i.e. below detection). In some cases, degradation can occur so fast that the ratio can go from 1 down to below detection between two measurement points. It is therefore quite important that the ratios below detection are not simply discarded. However, it is not appropriate to include many datapoints below detection in the degradation curve fit. It is rather advised to limit the datapoints included in biodegradation kinetic fitting to two datapoints below detection and discard any further datapoints.

#### Fitting biodegradation kinetic models

Biodegradation kinetics can be determined from the fit of a biodegradation kinetics model to the biodegradation peak area ratios. In simulation biodegradation testing low concentrations of test chemicals are used. At high initial density of relevant degraders, biodegradation kinetics will follow a first order degradation model [24] that is described by a first order degradation constant, k_first order_ (Eq. (2)).

(2) $$S=S_{0}\cdot e^{-k_{first\;order}t}$$

S is the concentration of the substrate (test chemical) as a function of time, t, S_0_ is the initial concentration of substrate.

At low initial density of relevant degraders, models are described by two fitted parameters because growth of the bacterial population influences the degradation kinetics. If the test chemical is assumed to be the sole substrate to support growth of the degraders, a logistic model can be used to describe the degradation (Eq. (3)) [24].

(3) $$S=\frac{S_{0}+X_{0}}{1+\frac{X_{0}}{S_{0}}e^{k_{logistic}\left(S_{0}+X_{0}\right)t}}$$

X_0_ is the amount of substrate required to produce the initial population density of the bacterial degraders responsible for the degradation of the test chemical, and k_logistic_ is the logistic rate constant (k_first order_ = k_logistic ·_ X_0_ [24]). It is not trivial to determine what fraction of the bacterial population that is responsible for the degradation of a certain chemical in a natural water inoculum. Even though the logistic model may be used to interpolate the X_0_, it should not be interpreted strictly, since adaptation processes as well as growth of the microbial population may influence the initial phase of the incubation.

In a simulation biodegradation test where multiple substrates are present, the processes are even more complicated, because the bacteria responsible for degradation of one of the test substances may grow on more than one substrate, and the growth is therefore not directly linked to the biodegradation of the test substance. In this case, co-metabolism models can be used. If exponential growth of the bacterial population is assumed, Eq. (4) can be used to describe the degradation of test substances at low concentrations [25].

(4) $$S=S_{0}\cdot e^{-\frac{k_{co-metabolism}}{r}\left(e^{r\cdot t}-1\right)}$$

*r* is the maximum specific growth rate in the particular environment and *k_co-metabolism_* is a rate constant that depends on the population density of the degraders, the maximum specific reaction rate of the degradation, and the half-saturation constant for the reaction.

**Fig. 3:**
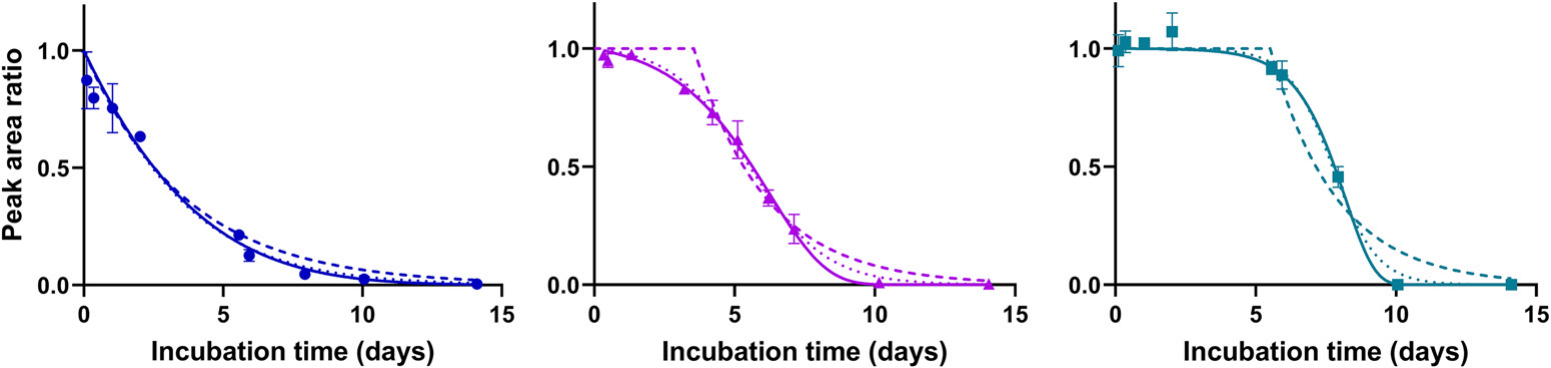
Fitting the co-metabolism model (solid lines), the logistic model (dotted lines) and the first order degradation with lag phase model (dashed lines) to three different datasets. The models are nearly identical for datasets without lag phase (left plot). Identification of the transition point between the lag phase and first order degradation phase is better with more data in the degradation phase.

A model including a lag phase followed by a first order degradation phase is often used as a practical approximation to the Eq. (3) or (4). The difference between the fit of a logistic model, a co-metabolism model, and a ‘lag phase + first order’ model is illustrated in Fig. 3. The fitting can be done by various computer programs, or alternatively, for the lag-phase + first order model it can be done using semi-logarithmic plots as described in the OECD 309 [2].

### Interpretation of results

#### Partitioning corrections for volatile chemicals

Persistence should be obtained based on test system biodegradation kinetics. However, volatile chemicals distribute highly towards the headspace in the test system, and if the degradation kinetics are used for fate modeling purposes, this partitioning can be taken into account to derive water phase biodegradation rate constants. If a headspace of 25% is chosen, then chemicals with a K_H_ above 10^−3^ atm m^3^ mol^−1^ would distribute with > 1% present in the headspace. The fraction present in the headspace for any combination of Henry's constants and headspace ratio, can be calculated from Eq. (5):

(5) $$f_{\mathrm{headspace}}=\frac{V_{\mathrm{headspace}}K_{H}}{V_{\mathrm{headspace}}K_{H}+V_{\mathrm{water}}\mathrm{RT}}$$

Where K_H_ is the Henry's laws constant [atm m^3^ mol^−1^], *V_water_* is the volume of the water phase [mL], *V_headspace_* is the volume of the headspace [mL], R is the gas constant [atm m^3^ *K* ^−^ ^1^ mol^−1^], and T is the temperature [K].

Assuming that bacteria responsible for degradation are present in the water phase, the headspace acts as a buffer for volatile chemicals [4]. This can be corrected for by the calculation of the degradation rate constant in the water phase (Eq. (6) [4]).

(6) $$k_{\mathrm{water}}=k_{\mathrm{system}}\frac{V_{\mathrm{water}}+K_{\mathrm{air},\mathrm{water}}V_{\mathrm{headspace}}}{V_{\mathrm{water}}}$$

Here *k_water_* is the first order biodegradation rate constant in the water phase [*d* ^−^ ^1^], *k_system_* is the first order biodegradation rate constant for the test system [*d* ^−^ ^1^] (as measured when evaluating biodegradation kinetics from decline of concentration of total amount of the test chemical), and *K_air,water_* is the air-water partition ratio (dimensionless and equal to K_H_/RT).

#### Ensuring high data quality

The fit of a biodegradation kinetic model to the results can be ambiguous. As discussed above one reason for this is that the biodegradation is caused by a complex interaction between mixed microbes and enzymes and not fully represented by the kinetic model. Another reason is that true replicate test systems are used, and therefore each test system may develop differently from the other test systems. A higher impact of this is seen if inocula with low bacterial numbers are used compared with inocula with high bacterial numbers [5]. A higher impact is also seen for chemicals that need adaptation of the microbial consortia and thus have longer lag phases compared with chemicals that are immediately degraded in the test [22].

Quality criteria can be set up to reveal three levels of information from the data. In most cases, data are sufficient for determining biodegradation kinetics or to categorize the test substance as not degraded. In some cases, the data are not sufficient to determine biodegradation kinetics, while it is still certain that the chemical was degraded in the test. A degradation half-time may then be reported (time to 50% removal by degradation). In rare cases, there may be single datapoints indicating degradation, but the data is not clear enough to draw any conclusion. After fitting the biodegradation kinetics model to the data, the fits should therefore be evaluated to determine whether the fits can appropriately describe the data. A first evaluation can be based on R^2^ being larger than e.g. 0.7 or 0.8 [5,14]. If less than 20% degradation is observed, the uncertainty between replicate test systems often renders it impossible to prove degradation, and the chemical should be categorized as not degraded. In the lag phase + first order degradation model, the transition point between the lag phase and the degradation phase can be uncertain if the time resolution on the data is not high around the transition point. For this model, an additional criterion, that at least two datapoints should be between 0.1 and 0.9 and thus be located on the degradation curve, can be added to determine the first order degradation rate constant. If complete degradation occurs between two datapoints, i.e., the biotic/abiotic ratio drops from >0.9 to <0.1, a minimum degradation rate constant can be determined based on the distance between the two datapoints. If more than two datapoints are in the range 0.1–0.9, but the R^2^ of the fit is less than 0.7, then information can still be obtained from the data. The degradation half-time can for example be read from the data, e.g., as the time for the first datapoint below a ratio of 0.5. If datapoints alternate between above and below 0.5 it may be necessary to categorize the degradation as ‘not conclusive’. If no datapoints are below 0.5 a degradation half-time should not be reported since this would be based on extrapolation outside of the experiment time and would be very uncertain.

When testing mixtures, it is much easier to get high quality data for the degradation half-times than for the first order rate constants as illustrated in a study with petroleum hydrocarbons [5], where biodegradation half-times were determined for 44 of 52 chemicals, but first order biodegradation half-lives only for 18 chemicals.

### Validity of the test

The validity of the test can be evaluated based on the removal of a reference substance as described in the OECD 309 test.

Additionally, valid biodegradation kinetic results for each test substance require (1) that biotic degradation is markedly faster than abiotic degradation and losses, (2) that the analytical QA/QC requirements are met and (3) that results based on regression fitting are cross checked against the actual measurements (as described in the section above).

### Test report

The test report should be based on the requirements detailed in the OECD 309 guidelines. Substantiated descriptions of the modifications to the test methods should also be supplied along with relevant references.

## Applications and improvements

Since this method was introduced in 2017, it has been used for various applications. An overview of these is listed in Table 1. These studies demonstrate the versatility of the method and the wide applicability domain in terms of chemicals space of difficult test chemicals (volatility, hydrophobicity, and mixtures/UVCBs), that are not testable with the current standard biodegradation testing guideline.

**Table 1:** Applications of the biodegradation test method.

Reference	Focus of study	Test chemical mixture	Addition method
[4]	Establish method for biodegradation testing of volatile chemicals	9 hydrocarbons: C9-C12, linear, cyclic, and aromatic	Passive dosing
[17]	The effect of inoculum origin on biodegradation kinetics	9 hydrocarbons: C9-C12, linear, cyclic, and aromatic	Passive dosing
[5]	Biodegradation kinetics of hydrocarbons covering a large chemical space	53 hydrocarbons: C8-C20, linear, branched, cyclic, and aromatic	Passive dosing
[13]	Mixture and concentration effects on biodegradation	1–16 hydrocarbons: C8-C19, linear, branched, cyclic, and aromatic	Passive dosing
[7]	Concentration effects on biodegradation kinetics of UVCBs	Diesel oil (UVCB) Lavender oil (UVCB)	Passive dosing
[8]	Whole substance testing of UVCBs yielding kinetics of constituents	Cedarwood oil (UVCB)	Microvolume spiking
[9]	The effect of incubation temperature on biodegradation kinetics	45 hydrocarbons: C6-C18, linear, branched, cyclic, and aromatic	Passive dosing
[18]	Linking incubation temperature and microbial composition	45 hydrocarbons: C6-C18, linear, branched, cyclic, and aromatic	Passive dosing
[14]	Concentration effect on biodegradation kinetics	Fragrances, plasticizers, UV filters and PAHs	Passive dosing
[22]	Mixture effect on biodegradation kinetics	Fragrances, flavoring, preservatives, and high boiling point solvents	Passive dosing

Table 2 shows an overview of the major testing challenges for volatile and hydrophobic chemicals in mixtures, and the modifications of the new biodegradation test method to address these challenges.

**Table 2:** Testing challenges and modifications made to address these.

Major testing challenges	Modifications relative to OECD 309
Avoiding evaporative losses of (semi)volatiles during test setup and incubation	**Liquid handling with gas tight syringes, incubation in gas tight analytical glass vials**
Highly volatile test substances partition into the headspace of test system	**Option included to calculate aqueous biodegradation kinetics based on test system dynamics and partitioning**
Avoiding test substance losses and contamination during sample treatment and analysis	**No manual sample treatment steps. Automated Solid Phase Microextraction coupled to GC–MS on unopened test system**
Evaporative and sorptive losses occurring even in closed, unopened analytical glass vials	**Accounting for moderate losses using peak area ratios (PAR) between test vials and abiotic controls. PAR account also for temporal changes in instrument sensitivity**
Ensuring aerobic conditions in a closed test system	**Headspace volume dimensioned to provide sufficient oxygen for microbial growth and biodegradation**
Co-solvent should be avoided/minimized (co-substrate, biological oxygen demand, biofilm formation)	**Test substances are introduced via passive dosing or microvolume spiking**
Inoculum differences between tests can confound comparisons of biodegradation between chemicals	**Testing multiple chemicals in mixtures gives better aligned data. Comparison between chemicals in mixture are not confounded by inoculum differences**
Simulation biodegradation testing is costly and time consuming (^14^C labeling, chemical by chemical testing)	**Testing non-labeled chemicals, 20–50 chemicals are tested simultaneously in a mixture**

Further improvements to the method could include modifications to allow the simulation biodegradation testing of hydrophobic and volatile chemicals in mixtures with the presence of suspended solids such as sediments or sludge.

## Funding

The novel biodegradation platform was developed and applied within research projects funded by Concawe, the Research Institute for Fragrance Materials (RIFM), CEFIC-LRI, Unilever and the Technical University of Denmark (DTU). The present paper summarizes the methodological developments, and its writing was supported by the company Global Product Compliance, (GPC) and DTU.

## CRediT authorship contribution statement

**Heidi Birch:** Conceptualization, Methodology, Investigation, Visualization, Writing – original draft. **Rikke Hammershøj:** Visualization, Writing – review & editing. **Mette Torsbjerg Møller:** Visualization, Writing – review & editing. **Philipp Mayer:** Conceptualization, Methodology, Writing – review & editing, Supervision, Funding acquisition.

## Declaration of Competing Interests

The authors declare that they have no known competing financial interests or personal relationships that could have appeared to influence the work reported in this paper.

## Data Availability

Data will be made available on request.
